# Outer Retinal Dysfunction on Multifocal Electroretinography May Help Differentiating Multiple Sclerosis From Neuromyelitis Optica Spectrum Disorder

**DOI:** 10.3389/fneur.2019.00928

**Published:** 2019-08-27

**Authors:** Thiago G. Filgueiras, Maria K. Oyamada, Rony C. Preti, Samira L. Apóstolos-Pereira, Dagoberto Callegaro, Mário L. R. Monteiro

**Affiliations:** ^1^Laboratory of Investigation in Ophthalmology (LIM 33), Division of Ophthalmology, University of São Paulo Medical School, São Paulo, Brazil; ^2^Department of Neurology, University of São Paulo Medical School, São Paulo, Brazil

**Keywords:** multiple sclerosis, neuromyelitis optica, electroretinogram, optical coherence tomography, retinal layers

## Abstract

**Purpose:** To evaluate the intermediate and outer retina of patients with multiple sclerosis (MS) and neuromyelitis optica spectrum disorder (NMOSD) using OCT and multifocal electroretinography (mf-ERG).

**Methods:** Patients with MS (*n* = 30), NMOSD (*n* = 30), and healthy controls (*n* = 29) underwent visual field (VF), OCT, and mf-ERG testing. The eyes were distributed into 5 groups: MS with or without history of ON (MS+ON, MS–ON), NMOSD with or without ON (NMOSD+ON, NMOSD–ON), and controls. The thickness of the macular retinal nerve fiber layer (mRNFL), ganglion cell layer (GCL), inner plexiform layer (IPL), inner nuclear layer, outer plexiform layer, outer nuclear layer, and photoreceptor layer was measured. mf-ERG P1 and N1 responses were registered and grouped in 3 sets of rings. The groups were compared using GEE models, and effect size (ES) calculated.

**Results:** Compared to controls, GCL and IPL thickness was significantly smaller in MS+ON (both *p* < 0.01), MS–ON (*p* < 0.01 and *p* = 0.015, respectively), NMOSD+ON (both *p* < 0.01) and NMOSD–ON (*p* = 0.03 and *p* = 0.018, respectively). ES was >0.80. mRNFL was smaller in three of the above groups (*p* < 0.01, *p* < 0.001, and *p* = 0.028; ES > 0.80) but not in MS–ON eyes (*p* = 0.18). No significant difference was observed for the remaining layers. Compared to controls, P1 and N1 peak times were shorter in MS (*p*-values in the range 0.049–0.002, ES < 0.50; and 0.049–0.010; ES < 0.50, respectively) but not in NMOSD. These abnormalities were strongly correlated with intermediate and outer retinal layer thickness.

**Conclusions:** mf-ERG data suggest outer retinal abnormalities in MS, but not in NMOSD. Our results may help understand how the two conditions differ regarding retinal damage.

## Introduction

Anterior visual pathway involvement is an important clinical manifestation of both multiple sclerosis (MS) and neuromyelitis optica (NMO) (1)—two autoimmune diseases which frequently lead to optic neuritis (ON) and/or transverse myelitis (2). ON is usually more severe in NMO than in MS. While the pathophysiology of the two conditions differ with regard to immune mechanisms, both lead to optic nerve damage and retrograde degeneration of the retinal nerve fiber layer (RNFL) and ganglion cell layer (GCL) (1–4).

With the discovery of the NMO-immunoglobulin G (IgG) autoantibody (5), it became clear that NMO and MS are different diseases (6). In NMO, the autoantigen target is aquaporin-4 (AQP4)—a water channel protein found primarily in astrocytes in the nervous system (e.g., in the optic nerve and spinal cord), but also in supporting cells like retinal Müller cells (7). The classic diagnostic criteria for NMO include a history of ON and acute transverse myelitis (8), but recently the definition was expanded to include clinical signs which allow to establish a diagnosis of NMO spectrum disorder (NMOSD) using an algorithm of revised diagnostic criteria (9). Thus, patients with isolated ON or LETM may now be diagnosed with NMOSD provided they test positive for anti-AQ4 antibody or have a specific combination of clinical and radiological findings (9, 10).

The quantification of retinal axonal loss in both MS and NMOSD has received much attention following the advent of spectral-domain optical coherence tomography (SD-OCT). It has been suggested that OCT-measured peripapillary RNFL (pRNFL) and macular thickness analysis may be useful in the monitoring of disease severity, and possibly in the differentiation of the two diseases (2). Both MS and NMOSD can present ON-related or subclinical abnormalities in pRNFL and macular thickness measurements (4, 11), but the two conditions may differ with regard to structural and functional retinal damage (2–4, 12, 13). Furthermore, segmented analyses of other retinal layers show that not only are the macular RNFL (mRNFL) and GCL reduced, but the inner nuclear layer (INL) may display abnormalities such as increased thickness and microcysts (3, 14, 15). As for the remaining layers, no study on NMOSD and only one study on MS (16) has specifically addressed the evaluation of the outer retinal layers separately using SD-OCT.

Electrophysiological testing is another important way of evaluating retinal function. One such method, pattern electroretinography, uses a temporally modulated patterned stimulus of constant mean luminance to assess the function of the inner retinal ganglion cells (12, 17). On the other hand, outer retinal function may be evaluated with full-field electroretinography (ERG) (used to measure global retinal response) and multifocal electroretinography (mf-ERG) (used to capture the response of specific areas in the central retina), mostly originating from photoreceptors and bipolar cells (18).

While damage to the inner retinal layer is well-documented in MS and NMOSD, the possibility of primary or secondary involvement of the outer retinal layers, including the photoreceptor layer (PRL), as recently hinted at by histological, OCT, and electrophysiological findings in patients with MS, remains to be clarified (3, 19–22). In MS, while some studies using OCT indicate primary involvement of the outer retinal layer in a subset of patients (21), others suggest outer retinal changes may be secondary to disease-related ON (23, 24). As for electrophysiological evaluations of the outer retina, no study on NMOSD and few studies on MS have used this approach (16, 25–27). Findings for MS patients vary significantly between studies and only one recent study has evaluated the correlation between ERG, mf-ERG, and OCT-measured outer retinal layers (16). Thus, more knowledge of the outer retinal structure and function in MS and NMOSD is needed, not only to understand the disease process but also to avoid diagnostic confusion with retinal disease.

The purpose of this study was therefore to investigate possible outer retinal involvement in eyes of patients with MS and NMOSD. To do so, we used mf-ERG and high-resolution OCT to evaluate the outer retinal layers in normal controls and in patients with MS or NMOSD (with and without history of ON) and tested for correlations between mf-ERG, OCT, and visual field (VF) measurements using standard automated perimetry (SAP). We also evaluated the relationship between mf-ERG and SD-OCT retinal thickness layer measurements.

## Methods

In this prospective, cross-sectional study, 110 eyes from 30 MS and 30 NMOSD patients, diagnosed based on previously described criteria (9, 28), and 29 controls were evaluated. Eyes were distributed into 5 groups: 1-MS with ON (MS+ON), 2-MS without ON (MS–ON), 3-NMOSD with ON (NMOSD+ON), 4-NMOSD without ON (NMOSD–ON), and 5-controls. Eighteen NMOSD patients tested positive for anti-AQP4 antibody. The study was approved by the Institutional Review Board Ethics Committee, followed the Declaration of Helsinki and informed consent was obtained from all subjects.

Disease duration, number of ON crises, and therapies were ascertained for each patient, by self-report and physician report, and confirmed by medical record review. The diagnosis of ON was established based on the history of acute progressing vision loss generally associated with pain on eye movement and documentation of decreased VA, VF defect, relative afferent pupillary defect, and a fundus examination showing either normal findings or optic disc edema. Patients with ON episodes <6 months before study entry were excluded since OCT-measured axonal loss after ON occurs up to 6 months. Other exclusion criteria were: central nervous system infectious disease, brain MRI abnormalities other than those of MS or NMOSD, and diabetes mellitus. The controls were normal healthy volunteers recruited from among companions of patients and hospital staff. Ophthalmic exclusion criteria for patients and controls were: (i) history of intraocular pressure elevation, (ii) optic neuropathies other than ON, (iii) clinical signs of glaucoma, (iv) retinal diseases, and (v) optic disc anomalies.

### Ophthalmic Examination

Subjects underwent complete ophthalmic examination, including best-corrected monocular visual acuity (VA) evaluation and SAP. VA was assessed with ETDRS charts at 3.2 m. Snellen equivalents were also recorded for ETDRS VA measurements. SAP was performed with a Humphrey Field Analyzer (Carl-Zeiss Meditec, Dublin, CA) using the 24-2 SITA-standard strategy, with a Goldmann size III stimulus.

Inclusion criteria for patients were: (i) VA of 20/200 or better in at least one eye, (ii) spherical refraction within ±3 D and cylinder refraction within ±3 D, (iii) intraocular pressure <21 mmHg, (iv) no concomitant ocular diseases, and (v) reliable VF (defined as one with fewer than 20% fixation losses, false-positive or false-negative responses). The inclusion criteria for controls were similar, except for VA and VF which had to be normal.

Each point tested on the VF represented the difference in luminance threshold (dB) between the study subjects and the age-matched normal value. The severity of VF defects was proxied by mean deviation (MD), i.e., the mean value of the data on the total deviation plot after excluding the two outer nasal points (totaling 50 points) in order to allow for a better match between VF and mf-ERG measurements. The deviation from normal at each test point was measured in dB (logarithmic scale) and converted into 1/Lambert (linear scale) by dividing the decibel value by 10 and unlogging the quotient. The deviation from the normal sensitivity of the central VF (central mean deviation—CMD) was obtained by averaging the mean deviation of the 12 central points on the 24-2 strategy VF test. The points selected for this parameter stimulate approximately the same area in the macular region analyzed by the circular ETDRS protocol of the OCT.

### Optical Coherence Tomography

Following pupil dilation with 1% tropicamide the subjects were submitted to OCT scanning (Spectralis® OCT, Heidelberg Engineering, Heidelberg, Germany) of the macular and peripapillary area.

The high-resolution scans (100 averaged scans) were aligned to the center of optic nerve head. For the macular area, scanning involved the acquisition of 61 horizontal B-scans (average of 16 frames each), covering a 30° × 25° (9.2 × 7.6 mm) area centered in the fovea (40,000 scans/s, axial resolution of 5 μm) that were acquired using the automated eye alignment-tracking software (TruTrack, Heidelberg Engineering, Heidelberg, Germany). Criteria for acceptable fundus images included: absence of large eye movements (defined as an abrupt shift completely disconnecting a large retinal vessel), consistent signal intensity level across the scan, and absence of black bands (caused by blinking) throughout the examination. Images were also required to have centered scans and a signal strength >20 dB and to comply with the previously described OSCAR-IB quality criteria (29).

**Figure 1:**
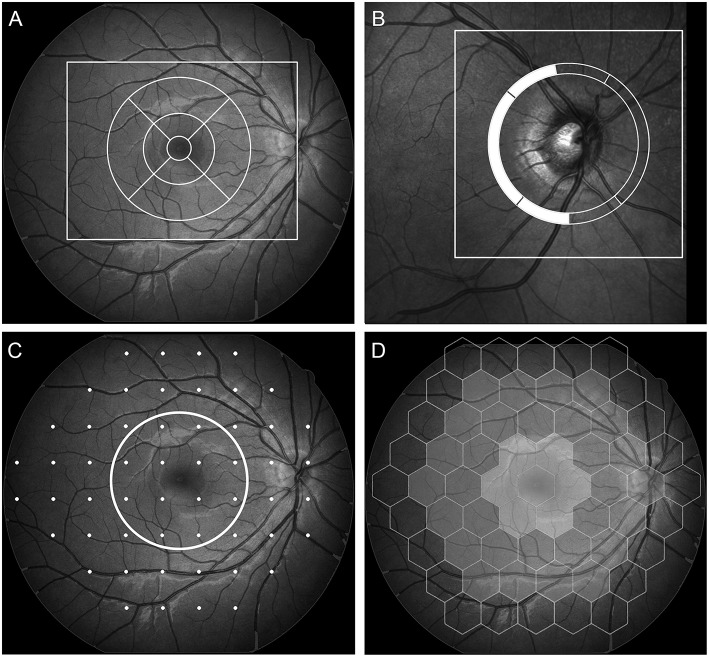
Above: demarcation of the areas (outer square) in the macula **(A)**, and optic nerve **(B)** scanned with spectral domain-OCT, with a schematic representation of the macular thickness map according to the ETDRS grid (**A**, circle) and peripapillary RNFL thickness according to a previously published division **(B)** highlighting the superior temporal, temporal, and inferior temporal segments averaged in the study. Below: demarcation points read on 24-2 standard automated perimetry **(C)**. The 12 points contained in the central circle correspond to the area evaluated in the circular macular map. Schematic view of the 61 multifocal electroretinographic hexagons divided into two central, two intermediate, and one outer (excluded from analysis) circles of stimulus **(D)**.

Macular thickness measurements were determined according to the ETDRS grid, with anatomical quadrants (superior, inferior, nasal, and temporal) of the inner (3 mm) and outer (6 mm) circles centered on the fovea (Figure 1A). pRNFL measurements were acquired in a circle of 1536 A-scan points subtending 12° centered on the optic disc with a diameter of 3.5 mm (Figure 1B). The temporal margin of the optic disk was chosen as a landmark and labeled 0°. From this point, the software divided the pRNFL into six sectors: temporal (310–41°), superotemporal (41–80°), superonasal (80–120°), nasal (121–230°), inferonasal (231–270°), and inferotemporal (271–310°), clockwise in the right eye and counterclockwise in the left eye. To better assess the correlation between pRNFL thickness measurements and mf-ERG parameters, we calculated the 170° corresponding to the average of the temporal, superotemporal and inferotemporal sector measurements (Figure 1B).

Macular scans were processed and submitted to automatic segmentation with the equipment's software. After segmentation, the thickness of the following layers was measured: mRNFL, GCL, inner plexiform layer (IPL), INL, outer plexiform layer (OPL), outer nuclear layer (ONL), and the PRL. The average thickness of each layer was calculated according to the ETDRS grid, with anatomical quadrants (superior, inferior, nasal and temporal) of the inner (3 mm) and outer (6 mm) circles centered on the fovea. Average thickness measurements were calculated as the weighted average of the sectoral thickness measurements, excluding the fovea.

### Electroretinography

mf-ERG recordings were made using the RETIscan System(Roland Consult, Wiesbaden, Germany), following the International Society for Clinical Electrophysiology of Vision guidelines (30). The pupils were previously dilated with 1% tropicamide eye drops. Tetracaine and methylcellulose eye drops were used for corneal electrode placement. The signals were captured with ERG-Jet contact lens electrodes.

The stimulus array used consisted of 61 hexagons scaled with a 4.0 eccentricity and distortion factor, presented on a 21-inch rectangular black-and-white flat screen monitor (CRT color monitor, Roland Consult) at a distance of 26 cm, in a semi-dark, acoustically isolated room. Directed at the central 30° of the retina, the stimulus was set to 97% contrast and a 60 Hz frame rate. The optical correction was adjusted to the distance of the stimulation screen and the patient was instructed to gaze fixedly at the point of intersection of two diagonal red lines crossing the entire stimulus screen. The test consisted of registering the responses to eight 47-s stimulation cycles filtered with a bandpass between 10 and 100 Hz, in addition to being amplified. Noise from blinking and eye movements was automatically ignored by the software. The analyses were made by evaluating the first-order kernel response peak time and amplitude of N1 (measured from the isoelectric line to first negative peak) and the peak time and amplitude of P1 (measured from the first negative peak, N1, to the first positive peak, P1). N1 and P1 responses were grouped in concentric rings. After excluding the outermost ring, we calculated the following measurements: the average response of rings 1 and 2 (the two inner rings, from 0 to 10 degrees), rings 3 and 4 (the two remaining outer rings, from 10 to 20 degrees) and all four rings (from 0 to 20 degrees) (31) (Figure 1D). The responses were grouped to facilitate comparisons and to ensure the mf-ERG data were closely matched with the fundus areas/points evaluated in the VF, the pRNFL and the OCT macular parameters (Figures 1C,D).

### Statistics

Descriptive statistics were expressed as mean values ± SD. The Shapiro-Wilk test was used to evaluate the normality assumption. Generalized estimating equation (GEE) models accounting for age, sex, and within-patient inter-eye correlations were used to compare the groups with regard to OCT, ERG, and mf-ERG findings. Effect size (ES) was evaluated with Cohen's *d* as the difference between the means of two groups divided by the standard deviation of the reference group. ES serves the purpose of further emphasizing the results in each comparison and was interpreted according to the classification: small 0.20–0.49; medium 0.50–0.79; large ≥ 0.80 (32). Pearson's correlation coefficients were used to assess potential associations between parameters. Analyses were performed with the software IBM SPSS Statistics V. 21.0. The level of statistical significance was set at 5% (*p* < 0.05).

## Results

A total of 30 patients (26 female) with MS and 30 (25 female) with NMOSD randomly selected from the outpatient clinic, and 29 healthy controls were included in the study. Demographic data of all individuals studied are shown in Table 1. Sixteen of the patients with MS had a history of ON (bilateral in 6 and unilateral in 10). Since no eye was excluded from the study, 22 eyes were classified as MS+ON and 38 as MS–ON. Of the 30 MS patients, 23 had relapsing-remitting, 5 primary-progressive, and 2 secondary-progressive form of the disease. Disease duration in MS patients without ON was 7.29 ± 4.87 years and in MS patients with ON was 10.00 ± 6.90 years. The mean number of ON attacks (± SD) in eyes of patients with MS was 1.31 ± 0.64. The time period between the single or the last ON attack and the study in MS patients was 4.47 ± 5.23 years. Twenty-one of the 30 patients with NMOSD had a history of ON (bilateral in 12 and unilateral in 9). Ten of the 60 eyes of NMOSD patients with history of ON were excluded because VA was worse than 20/200. Of the 50 eyes that remained in the study, 23 had a history of ON and 27 did not. Disease duration in NMOSD patients without ON was 4.33 ± 4.27 years and in those with ON was 7.21 ± 6.66 years. The mean number of ON attacks (±SD) in eyes of patients NMOSD was 1.52 ± 1.03. The time between the single or the last ON attack and the study in NMOSD patients was 4.60 ± 4.27 years. Among the patients with MS 18 were treated with interferon, 4 with glatiramer acetate, 2 with natlizumab, 1 with methotrexate, 3 with combined therapy with interferon and natalizumab, and 1 with interferon and glatiramer acetate. Among NMOSD patients, 9 were on Azathioprine monotherapy, 3 on Prednisone, 3 on Rituximab, 1 on Cyclophosphamide, and 14 on combined therapy with azathioprine and prednisone. Six had received plasmapheresis at some stage in the course of the disease and one intravenous immunoglobulin. The control group included 57 eyes of 29 subjects. One eye of the control group was excluded due to reduced VA caused by an epiretinal macular membrane. VF MD and CMD were significantly lower in MS+ON (*p* < 0.001; ES = 1.47) and NMOSD+ON (*p* < 0.001; ES = 1.10) than in controls. MD and CMD was lower in eyes affected with ON.

**Table 1:** Demographic characteristics, visual acuity and visual field data of patients with Neuromyelitis Optica Spectrum Disease (NMOSD) and Multiple Sclerosis (MS) with and without optical neuritis (ON) and normal controls.

**Group**	**MS**		**NMOSD**		**Controls**
Subjects	30		30		29
Eyes excluded from the study	None		10		1
Gender M/F	4/26		5/25		9/20
Eyes studied	MS–ON (*n* = 38)	MS+ON (*n* = 22)	NMOSD–ON (*n* = 27)	NMOSD+ON (*n* = 23)	Controls (*n* = 57)
Disease duration, years (SD)	7.29 ± 4.87	10.00 ± 6.90	4.33 ± 4.27	7.21 ± 6.66	–
Age, years, mean (SD)	36.76 (8.82)	36.53 (12.44)	38.69 (12.90)	35.03 (11.14)	45.37 (10.58)
MD in dB, mean (SD)	−4.00 (0.93)^*^^δ^	−6.27 (1.28)^*^	−2.06 (0.42)^δ^	−9.03 (2.23)^*^	−1.23 (0.26)
CMD in dB, mean (SD)	−3.20 (0.8)^*^^δ^	−4.65 (0.88)^*^	−1.82 (0.31)^δ^	−7.55 (2.20)^*^	−1.22 (0.22)

SD, Standard deviation; MS–ON, MS eyes without ON; MS+ON, MS eyes with ON; NMOSD–ON, NMOSD eyes without ON; NMOSD+ON, NMOSD eyes with ON; VA,visual acuity; MD, mean deviation; CMD, central mean deviation.

* P < 0.05 compared with controls;

δ *P < 0.05 compared with corresponding ON groups; Generalized Estimating Equations (GEE) models*.

After applying the inclusion and exclusion criteria, the final sample consisted of 167 eyes distributed as follows. MS–ON: 38 eyes; MS+ON: 22 eyes; NMOSD–ON: 27 eyes; NMOSD+ON: 23 eyes; controls: 57 eyes. Thirteen of the 21 patients with NMOSD and ON were positive for anti-AQP4 antibody. Nine NMOSD patients had LETM but not ON; five of these were positive for anti-AQP4 antibody.

OCT-measured macular parameters for each retinal layer as well as pRNFL thickness measurement in all groups of eyes in presented in Table 2. The average mRNFL was significantly lower in eyes with history of ON than in controls. The mean pRNFL and mean macular GCL and IPL were also lower in MS and NMOSD eyes than in controls. No significant difference was observed when MS and NMOSD eyes with and without ON were compared to controls with regard to INL, OPL, ONL, and PRL. mRNFL, GCL, and IPL measurements in MS+ON and NMOSD+ON were smaller than in MS–ON and NMOSD–ON (ES > 0.80). The same was true for pRNFL, except that MS+ON and NMOSD–ON did not differ significantly (*p* = 0.20). Mean INL values were significantly higher in NMOSD+ON than in MS–ON (*p* = 0.01, ES = 0.73) or NMOSD–ON (*p* = 0.03; ES = 0.44).

**Table 2:** Mean values (± standard deviation) of Optical Coherence Tomography (OCT, in micrometers) of patients with multiple sclerosis (MS) and Neuromyelitis Optica Spectrum Disease (NMOSD) with (MS+ON, NMOSD+ON) and without (MS–ON, NMOSD–ON) history of optic neuritis (ON), and controls.

**OCT**	**MS–ON**	**MS+ON**	**NMOSD–ON**	**NMOSD+ON**	**Controls**	**MS–ON vs.** **MS+ON**	**MS–ON vs.** **NMOSD–ON**	**MS–ON vs.** **NMOSD +ON**	**MS+ON vs.** **NMOSD–ON**	**MS+ON vs.** **NMOSD+ON**	**NMOSD–ON vs.** **NMOSD+ON**
**Macula**											
RNFL	29.83 ± 5.08	25.19 ± 7.56^**^	28.97 ± 4.17	25.11 ± 7.82^**^	31.15 ± 4.26	*P = 0.003*	*P* = 0.46	*P = 0.01*	*P = 0.04*	*P* = 0.97	*P = 0.01*
GCL	39.46 ± 5.91^**^	34.37 ± 8.20^**^	39.62 ± 6.16^*^	33.06 ± 11.37^**^	42.48 ± 4.11	*P = 0.01*	*P* = 0.91	*P = 0.01*	*P = 0.01*	*P* = 0.66	*P = 0.004*
IPL	33.00 ± 4.62^*^	29.20 ± 6.13^*^	32.85 ± 4.40^*^	29.11 ± 6.56^*^	35.04 ± 2.87	*P = 0.01*	*P* = 0.69	*P = 0.01*	*P = 0.02*	*P* = 0.96	*P = 0.003*
INL	34.62 ± 2.59	35.38 ± 2.84	35.40 ± 2.77	36.77 ± 3.46	35.40 ± 2.97	*P* = 0.13	*P* = 0.25	*P = 0.01*	*P* = 0.98	*P* = 0.14	*P = 0.03*
OPL	30.55 ± 2.60	29.86 ± 2.38	29.88 ± 3.10	29.50 ± 2.82	29.60 ± 3.41	*P* = 0.23	*P* = 0.36	*P* = 0.15	*P* = 0.98	*P* = 0.64	*P* = 0.62
ONL	64.00 ± 7.75	65.23 ± 6.99	63.61 ± 7.38	62.14 ± 6.67	62.52 ± 9.39	*P* = 0.12	*P* = 0.84	*P* = 0.33	*P* = 0.43	*P* = 0.13	*P = 0.04*
PRL	79.58 ± 2.21	79.71 ± 1.96	79.63 ± 2.08	79.11 ± 1.53	79.31 ± 3.58	*P* = 0.70	*P* = 0.92	*P* = 0.32	*P* = 0.90	*P* = 0.25	*P* = 0.23
**Optic nerve**											
RNFL (170°)	98.37 ± 16.88^*^	86.29 ± 22.72^**^	93.24 ± 24.40^**^	75.77 ± 36.21^*^	107.34 ± 19.18	*P = 0.03*	*P* = 0.34	*P = 0.005*	*P* = 0.31	*P* = 0.24	*P* = 0.05

* P < 0.05

** *P < 0.01, compared to controls, significant P-values (within groups comparisons) in italics; Covariables: age (39.6 years) and sex. RNFL, macular retinal nerve fiber layer; GCL, ganglion cell layer; IPL, inner plexiform layer; INL, inner nuclear layer; OPL, outer plexiform layer; ONL, outer nuclear layer; PRL, photorreceptor layer; pRNFL, peripapillary retinal nerve fiber layer*.

**Figure 2:**
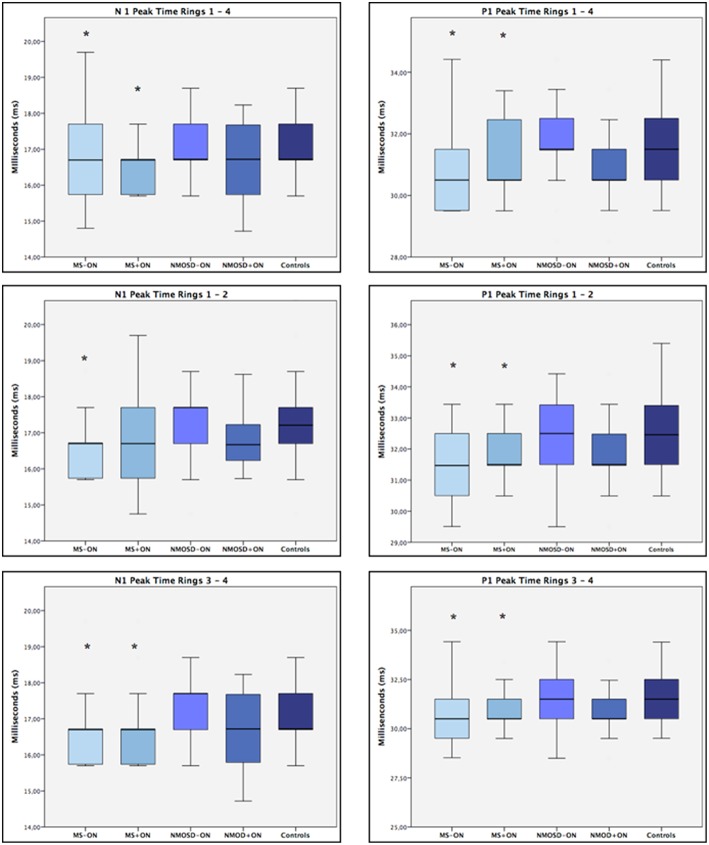
Boxplots of mean MF-ERG N1 and P1 peak times in milliseconds (ms) for controls and the four groups of patients. MS–ON = eyes with multiple sclerosis (MS) but no history of optic neuritis (ON); MS+ON = eyes with MS and history of ON; NMOSD–ON = eyes with neuromyelitis spectrum disease (NMOSD) but no history of ON; NMOSD+ON = eyes with NMOSD and history of ON. Median values and interquartile ranges (IQR) are indicated by horizontal lines and boxes, respectively; whiskers show the lowest and highest data points within the lower and upper quartiles. Note that mean values are slightly lower for MS than for controls, except for patients with history of ON when only the inner rings (1 and 2) are considered. *p < 0.05 (GEE).

Table 3 shows mf-ERG measurement results. No significant difference in N1 or P1 amplitude measurements was found between the groups, but significantly reduced peak time measurements (*p* < 0.05) were found in MS+ON and MS–ON with regard to all-ring average, outer ring average (mean N1 and P1), and inner ring average (mean P1). Significant *p*-values were in the range 0.049–0.002 and 0.049–0.010 for P1 and N1 peak times, respectively. The ES of the observed differences was small (ES < 0.5). No significant difference was found in any measurements in NMOSD patients, with or without ON. Figure 2 shows the relevant electrophysiological test data (mf-ERG peak times).

**Table 3:** Mean values (± standard deviation) found in multifocal electroretinogram (mf-ERG) of patients with multiple sclerosis (MS) and neuromyelitis optica spectrum disease (NMOSD), with (MS+ON, NMOSD+ON) or without (MS–ON, NMOSD–ON) history of ON and controls.

**mf-ERG**	**MS–ON**	**MS+ON**	**NMOSD–ON**	**NMOSD+ON**	**Controls**
**AVERAGE MEASUREMENTS OF RINGS 1–4**					
N1 amplitude	0.45 ± 0.12	0.41 ± 0.07	0.40 ± 0.11	0.40 ± 0.09	0.42 ± 0.10
N1 peak time	16.7 ± 1.18^*^	16.5 ± 1.03^*^	17.1 ± 0.87	16.9 ± 0.72	17.2 ± 1.16
P1 amplitude	1.82 ± 1.06	1.62 ± 0.64	1.54 ± 0.40	1.57 ± 0.49	1.57 ± 0.44
P1 peak time	31.0 ± 1.67^*^	30.9 ± 1.43^*^	31.5 ± 1.46	31.6 ± 1.55	31.7 ± 1.58
**AVERAGE MEASUREMENTS OF RINGS 1 AND 2**					
N1 amplitude	0.47 ± 0.10	0.46 ± 0.16	0.44 ± 0.13	0.42 ± 0.18	0.47 ± 0.15
N1 peak time	16.6 ± 0.93^*^	16.8 ± 1.06	17.1 ± 0.99	16.9 ± 0.83	17.3 ± 1.45
P1 amplitude	1.67 ± 0.52	1.61 ± 0.57	1.61 ± 0.50	1.48 ± 0.61	1.63 ± 0.54
P1 peak time	31.6 ± 1.33^**^	31.9 ± 1.03^**^	32.2 ± 1.34	32.4 ± 1.46	32.6 ± 1.55
**AVERAGE MEASUREMENTS OF RINGS 3 AND 4**					
N1 amplitude	0.44 ± 0.11	0.41 ± 0.07	0.40 ± 0.09	0.41 ± 0.10	0.41 ± 0.13
N1 peak time	16.6 ± 0.96^*^	16.7 ± 0.99^*^	17.2 ± 1.03	17.0 ± 0.74	17.1 ± 1.16
P1 amplitude	1.63 ± 0.41	1.61 ± 0.50	1.56 ± 0.44	1.54 ± 0.56	1.61 ± 0.40
P1 peak time	31.0 ± 1.64^*^	30.8 ± 1.03^**^	31.4 ± 1.51	31.6 ± 1.60	31.6 ± 1.67

Amplitude data in microvolts (μV) and peak time in milliseconds (ms).

* P <0.05

** *P <0.01, compared to controls; covariables: age (39.6 years) and sex*.

To evaluate whether morphofunctional impairment could have influenced the observed psychophysical responses, including VF and VA, we divided ON eyes of both MS and NMOSD patients into four subgroups according to baseline pathology and VA recovery (complete: VA = 20/20 vs. partial: VA < 20/20). Recovery among MS patients was complete for 18 eyes (“MS 20/20 eyes”) and partial for 4 (“MS<20/20 eyes”), while recovery among NMOSD patients was complete for 16 eyes (“NMOSD 20/20 eyes”) and partial for 7 (“NMOSD < 20/20 eyes”). The results of the comparisons regarding OCT-measured outer retinal layers, mf-ERG and VF parameters are presented in Table 4. No significant differences were found between thickness averages of outer layers when all groups were compared. Compared to controls, a significant reduction in N1 and P1 peak times was observed in all measurements of “MS 20/20 eyes,” except for P1 peak time in the analysis of all-ring average, though the difference came close to statistical significance (*p* = 0.067). A significant reduction in P1 amplitude was observed only in measurements of “MS < 20/20 eyes.” The two NMOSD subgroups (complete vs. partial recovery) did not differ with regard to mf-ERG parameters. Mean MD and CMD values were significantly lower for patients than for controls, especially in the partial recovery subgroups.

**Table 4:** Mean values (± standard deviation) of Optical Coherence Tomography (OCT) outer retinal layers, multifocal electroretinogram (mf-ERG), and visual field (VF) of patients with multiple sclerosis (MS) and neuromyelitis optica spectrum disorder (NMOSD), with history of ON and complete visual acuity recovery (20/20) or partial visual acuity recovery (<20/20) as well as controls.

	**MS 20/20**	**MS < 20/20**	**NMOSD 20/20**	**NMOSD < 20/20**	**Controls**
**OCT**					
OPL	29.87 ± 2.29	30.02 ± 3.37	29.26 ± 2.83	29.32 ± 1.93	29.60 ± 3.35
ONL	64.04 ± 8.17	68.36 ± 10.17	61.54 ± 7.28	62.72 ± 4.82	62.52 ± 9.39
PRL	79.60 ± 2.33	79.85 ± 1.63	79.06 ± 1.60	78.64 ± 0.71	79.31 ± 3.57
**Mf-ERG**					
**Average measurements of rings 1–4**					
N1 amplitude	0.42 ± 0.11	0.42 ± 0.07	0.42 ± 0.08	0.36 ± 0.10	0.42 ± 0.09
N1 peak time	16.50 ± 1.02^*^	17.27 ± 1.03	16.75 ± 0.89	17.06 ± 0.48	17.17 ± 1.16
P1 amplitude	1.67 ± 0.50	1.33 ± 0.58^*^	1.59 ± 0.44	1.39 ± 0.57	1.57 ± 0.40
P1 peak time	31.07 ± 1.31	31.58 ± 1.40	31.14 ± 1.55	31.25 ± 0.83	31.74 ± 1.59
**Average measurements of rings 1 and 2**					
N1 amplitude	0.50 ± 0.18	0.41 ± 0.26	0.46 ± 0.13	0.37 ± 0.20	0.47 ± 0.17
N1 peak time	16.66 ± 1.20^*^	17.57 ± 1.55	16.75 ± 0.88	16.98 ± 0.66	17.26 ± 1.45
P1 amplitude	1.70 ± 0.66	1.42 ± 0.50^*^	1.61 ± 0.43	1.29 ± 0.71	1.63 ± 0.51
P1 peak time	31.87 ± 1.09^*^	32.40 ± 0.94	32.03 ± 1.56	32.13 ± 0.74	32.57 ± 1.55
**Average measurements of rings 3 and 4**					
N1 amplitude	0.42 ± 0.14	0.43 ± 0.08	0.42 ± 0.09	0.36 ± 0.12	0.41 ± 0.10
N1 peak time	16.58 ± 1.04^*^	17.65 ± 1.63	16.81 ± 0.93	17.06 ± 0.54	17.13 ± 1.17
P1 amplitude	1.67 ± 0.58	1.34 ± 0.13^*^	1.64 ± 0.44	1.35 ± 0.63	1.61 ± 0.44
P1 peak time	30.94 ± 1.22^*^	31.60 ± 1.42	31.08 ± 1.57	31.43 ± 0.82	31.65 ± 1.66
**VISUAL FIELD**					
MD (dB)	−6.40 ± 5.73^*^	−21.73 ± 10.21^*^	−5.03 ± 4.51^*^	−22.52 ± 8.10^*^	−1.00 ± 1.56
CMD (dB)	−4.03 ± 2.42^*^	−21.44 ± 9.68^*^	−3.04 ± 3.23^*^	−20.87 ± 9.79^*^	−0.92 ± 1.34

* *P < 0.05 compared to controls; covariables: age (40.95 years) and sex. OPL, outer plexiform layer; ONL, outer nuclear layer; PRL, photoreceptor layer; MD, mean deviation; CMD, central mean deviation. Values of OCT data are in micrometers, mf-ERG amplitude in microvolt, peak time in milliseconds and visual field in decibels*.

Table 5 shows the relationship between OCT or VF parameters and mf-ERG peak time parameters in MS or NMOSD eyes. In MS eyes, significant negative correlations were found between N1 and P1 peak times (all-ring average and inner ring average) and INL thickness (−0.26 to −0.33) and between the N1 peak time (all-ring average) and ONL thickness (−0.26). In NMOSD eyes, a significant correlation was found between N1 peak time (inner ring average) and INL thickness (−0.26). The only significant correlation in relation to the VF was a negative correlation between N1 peak time (inner ring average) and CMD (−0.29) and between P1 peak time (inner ring average) and MD (−0.29) in MS eyes.

**Table 5:** Relationship between mf-ERG peak time parameters and OCT-measured macular layers, peripapillary RNFL and visual field sensitivity of patients in patients with multiple sclerosis (MS, *n* = 60) and neuromielitis optica spectrum disease (NMOSD, *n* = 50).

	**mfERG average of rings 1–4**				**mfERG average of rings 1 and 2**			
	**N1 peak time**		**P1 peak time**		**N1 Peak time**		**P1 peak time**	
	**MS**	**NMOSD**	**MS**	**NMOSD**	**MS**	**NMOSD**	**MS**	**NMOSD**
**OCT**								
FT	−0.13	0.10	0.08	0.17	−0.08	0.08	0.08	0.28
RNFL	0.23	−0.01	0.30	0.01	0.16	0.06	0.23	0.14
GCL	−0.09	0.04	0.06	0.05	−0.08	0.05	−0.01	0.17
IPL	−0.14	0.06	−0.01	0.11	−0.10	0.08	−0.08	0.22
INL	–**0.26**	−0.17	–**0.27**	−0.12	–**0.33**	–**0.30**	–**0.27**	−0.12
OPL	−0.11	0.06	−0.08	0.13	−0.06	0.15	−0.12	0.19
ONL	–**0.26**	0.07	−0.04	0.03	−0.04	−0.06	0.14	−0.02
PRL	0.20	0.03	0.15	0.15	0.12	0.02	0.08	0.18
pRNFL	−0.09	−0.08	0.01	−0.03	−0.20	−0.02	−0.05	0.13
**Visual Field (1/Lambert)**								
MD	−0.15	0.04	−0.22	0.09	−0.16	0.22	–**0.29**	0.19
CMD	−0.21	0.02	−0.16	0.09	–**0.29**	0.16	−0.23	0.19

*Data are Pearson's correlation coefficients: bold, p < 0.05; RNFL, macular retinal nerve fiber layer; GCL, ganglion cell layer; IPL,inner plexiform layer; INL, inner nuclear layer; OPL, outer plexiform layer; ONL, outer nuclear layer; PRL, photorreceptor layer; pRNFL, peripapillary retinal nerve fiber layer; MD, mean deviation; CMD, central mean deviation*.

## Discussion

In this study, our primary interest was to investigate particularly the outer retinal layers in MS and NMOSD eyes using both OCT and mf-ERG parameters. While our OCT data confirmed the occurrence of reduced inner retinal parameters in MS and NMOSD eyes, widely documented in the literature (3, 4, 22), no significant difference was observed between controls and MS or NMOSD patients with regard to OCT-measured thickness of the outer retinal layers. On the other hand, our mf-ERG findings did not show abnormality in NMOSD eyes, but an excitatory pattern was observed in both MS+ON and MS–ON, with reduced N1 and P1 peak time responses compared to controls (Table 1). Eyes with history of ON were further studied in a separate analysis, which revealed a reduction in peak time in MS eyes with completely recovered VA (20/20), but not in eyes with partial recovery (Table 2), when compared to controls. Reduction in P1 amplitude measurements were observed only when the group of 4 MS+ON eyes that did not recover 20/20 vision was compared to normal.

Previous studies indicate that mf-ERG responses are largely generated by bipolar retinal cells (18, 33) and may therefore be affected by damage to such cells. In such cases mf-ERG responses are generally characterized by amplitude reduction or prolonged peak time measurements. However, findings from monkey studies (18) suggest that peak times may also decrease under conditions that would lead to hyperexcitability of the bipolar on-cells and blockade or damage of the bipolar off-cells. Also, transiently reduced mf-ERG-measured peak times have been described in diabetic patients with hyperglycemia (34) and acute retinal changes from smoking (35) and alcohol (36). Researchers have attributed such abnormalities to changes in retinal oxygen consumption—either increased supply (i.e., hyperglycemia) (37) or increased consumption (i.e., nicotine or alcohol effect) (38)—resulting in augmented tissue blood flow (39), upregulated retinal metabolism and, consequently, hyperstimulation.

Excitatory ERG abnormalities may also occur in inflammatory retinal conditions. Ikeda et al. (40) reported supranormal ERG findings in the early stages of inflammatory ocular diseases, despite the absence of fundoscopic abnormalities. The authors hypothesized that the observed electrophysiological changes might be caused by an excess of extracellular glutamate—a neurotransmitter apparently involved in the pathogenesis of MS (41, 42). Therefore, the excitatory mf-ERG abnormalities found in our MS patients may reflect a state of a subclinical inflammatory retinal process intrinsic to the condition. Green et al. (19) performed histologic and immunohistochemistry examination of the retina of 82 patients with MS and found evidence of retinal inflammation and atrophy even irrespective of disease duration. Abnormalities were found not only in the RNFL and GCL but also in the INL, suggesting a widespread retinal inflammatory injury.

The finding of reduced peak time without other mf-ERG abnormalities in MS patients supports the existence of with retinal inflammatory changes in MS patients. It is conceivable that inflammation leads to hyperexcitability of the bipolar on-cells or damage of the bipolar off-cells, as suggested by animal studies (18), resulting in reduced peak time measurements. Peak time reduction suggests external retinal involvement at the level of photoreceptors and/or bipolar cells. Also, the observation that peak time measurements correlated significantly with INL and ONL thickness measurements on OCT (Table 5) supports the concept of retina involvement in MS. On the other hand, when a small subgroup of 4 “MS < 20/20 eyes” was analyzed in separate, no peak time reduction was observed, and P1 amplitude was reduced (Table 4), suggesting that damage to the bipolar on-cells, presumably in more advance state of the disease, may also occur leading to mf-ERG amplitude reduction.

Previous studies have either failed to show significant ERG abnormalities in MS patients (43, 44), or reported ERG amplitude reduction or prolonged peak time measurements (25, 27, 45) and the subject is clearly still poorly defined. Most those studies used full-field ERG and only two evaluated MS patients using mf-ERG, a technique that evaluates a more localized retinal response (compared to full-field ERG) and can help better understand the retinal abnormalities in the disease. In one study, Hanson et al. (16) found evidence of outer retinal changes in patients with MS. However, contrasting with our data, the only mf-ERG abnormality was a prolonged P1 peak time in MS patients. No OCT abnormalities in relation to the normative database were identified, but the authors did find thinning of the RNFL, the GCL+IPL complex and the INL in eyes with MS+ON, compared to MS–ON eyes. No associations between OCT layers and mf-ERG or peak time changes were identified. However, the authors used the normative database of the device for comparison rather than a control group. This is not as reliable as a controlled study with a healthy control group subjected to the same conditions of data acquisition and evaluation. Another study evaluating MS patients on mf-ERG was suggestive of primary retinal abnormalities (reduced mf-ERG P1 amplitudes) (21), but the sample was small (*n* = 7) and consisted of patients with a characteristic finding of macular thickness reduction despite normal pRNFL thickness measurements on OCT.

To our knowledge, our study is the first to identify excitatory retinal changes in MS. While we do not have an exact explanation for the fact that our findings differ from previous studies with regard to MS, they may reflect a specific type of the primary retinal disease process. MS and NMOSD have clearly heterogeneous pathophysiological and clinical spectra, with variations in presentation, types of involvement, duration and treatment. The observed differences between eyes with MS and NMOSD are further evidence of the different pathophysiological mechanisms determining the two conditions.

One important limitation of our study is the relatively small number of subjects enrolled. In addition, the subjects of the three groups (MS, NMOSD, controls) were not perfectly age-matched. However, we attempted to compensate for this limitation by using GEE (46), making it possible to analyze both eyes of many patients and controls (compensating for the intra-subject correlation) and to adjust for age and sex differences in each group. Another limitation is that we cannot rule out the existence of asymptomatic ON in our patients. Finally, because this was an exploratory study, *p*-value correction for multiple tests was not performed, potentially increasing the risk of false positive results. Therefore, further investigations are necessary to confirm our findings.

In conclusion, the current study confirms OCT abnormalities in the inner retina of both MS and NMOSD, and provides evidence of dysfunction in the outer retina in MS but not NMOSD patients. The finding of reduced peak time values in MS patients raises the hypothesis of an inflammatory retinal process producing hyperstimulation. While the pathophysiology of retinal changes in patients with demyelinating diseases remains unclear, our data suggest that detailed electrophysiological evaluations may be useful for a better understanding of the pathophysiology of these diseases and also points to the need for further studies on the subject.

## Data Availability

The datasets generated for this study are available on request to the corresponding author.

## Author Contributions

TF, MO, and MM: design of the study. TF, SA-P, and DC: patients examination and selection for the study. MO, DC, and MM: providing resources for the study. TF, MO, RP, SA-P, and MM: analysis of data and interpretation. TF, MO, RP, and MM: drafting of the paper. TF, MO, RP, SA-P, DC, and MM: review of the paper and final approval of the manuscript.

### Conflict of Interest Statement

The authors declare that the research was conducted in the absence of any commercial or financial relationships that could be construed as a potential conflict of interest.

## Abbreviations

CMD: central mean deviation
ES: effect size
GCL: ganglion cell layer
GEE: generalized estimated equations
INL: inner nuclear layer
MD: mean deviation
mRNFL: macular retinal nerve fiber layer
MS: multiple sclerosis
NMOSD: neuromyelitis spectrum disorder
ONL: outer nuclear layer
OPL: outer plexiform layer
PRL: photoreceptor layer
RNF: retinal nerve fiber layer
SAP: standard automated perimetry
SD-OCT: spectral domain-optical coherence tomography
SITA: swedish interactive thresholding algorithm
VA: visual acuity
VF: visual field.
